# Artificial Intelligence in Liver Transplantation: Clinical Applications, Challenges, and Future Directions

**DOI:** 10.3390/jpm16100501

**Published:** 2026-09-27

**Authors:** Sourav Choudhury, Emir Hoti, Vinay Kumar Kapoor

**Affiliations:** 1 Department of HPB and Liver Transplantation, St. Vincent’s University Hospital, D04T6F4 Dublin, Ireland; e.hoti@svhg.ie; 2 Deapartment of Surgical Gastroenterology, Mahatma Gandhi Medical College & Hospital (MGMCH), Mahatma Gandhi University of Medical Sciences & Technology (MGUMST), Jaipur 302022, India; vkkapoor@mgumst.org

**Keywords:** artificial intelligence, machine learning, liver transplantation, clinical decision support, graft survival, immunosuppression

## Abstract

Liver transplantation remains a technically demanding procedure despite continued surgical advances. Successful outcomes depend on balancing donor selection with perioperative complexity, where each decision shapes graft and patient survival. Conventional scoring systems such as Model for End-Stage Liver Disease (MELD)-based prioritisation only partially explain differences in patient outcomes across transplant centres worldwide. Advances in artificial intelligence (AI) and machine learning (ML)—including longitudinal modelling of electronic health records, deep learning for imaging, and multimodal data integration—offer new opportunities to individualise risk stratification for graft selection, predict allograft dysfunction and rejection, and improve long-term survival. Clinically developed AI models may strengthen organ matching decisions and acceptance, while identifying candidates who require intensified immunosuppressant monitoring or targeted diagnostic testing in the postoperative period, without increasing rejection risk. However, introducing AI/ML systems requires thorough validation, careful calibration, and ongoing clinical audit to reduce bias, detect technical errors, and ensure reproducible performance. This review summarises current and emerging clinical applications of AI in liver transplantation and explores near-future directions. These include AI-driven graft evaluation during organ retrieval and normothermic machine perfusion, along with emerging evidence for the safety, reliability, and clinical utility of AI in transplantation practice.

## 1. Introduction

Artificial intelligence (AI) and machine learning (ML) are increasingly used for prediction, imaging interpretation, and clinical decision support in medicine. Liver transplantation (LT) is well suited to these approaches because outcomes depend on numerous factors, including donor quality, donor–recipient matching, perioperative risk, and long-term graft function. AI/ML may improve risk stratification, allocation decisions, intraoperative planning, and personalised post-transplant care, particularly in adult LT cohorts, where most available evidence has been generated. However, paediatric validation remains limited and should not be inferred from adult studies without dedicated evaluation. Modern transplant programmes generate large volumes of laboratory values, physiological measurements, donor and graft data, imaging results, pathology, and molecular information. The true clinical benefit of these advanced models emerges when they undergo external validation, are linked to clear clinical actions such as increased monitoring or biopsy, and are sufficiently interpretable to guide allocation and post-transplant decisions. This review summarises AI/ML applications across the transplant pathway—including imaging, digital pathology, graft viability assessment, waitlist mortality, donor–recipient matching, and complication prediction—and outlines key barriers to clinical implementation.

## 2. Fundamental Technologies and Methodology

AI/ML methods used in transplantation include supervised and unsupervised algorithms such as XGBoost, artificial neural networks (ANNs), random survival forests, graph neural networks, and ensemble models. XGBoost, in particular, has shown strong performance in predicting transplant outcomes [1]. Random survival forests support post-transplant survival modelling, while deep learning and graph neural networks can capture complex immunological features and donor–recipient compatibility patterns. For example, graph neural networks can analyse immunological and biological compatibility factors to refine organ suitability assessments beyond traditional matching [2]. Integrative modelling is also crucial: combining donor age, renal and liver function, cause of death, type of donation, cold ischaemia time, recipient age, and comorbidities has improved outcome prediction [2]. Figure 1 outlines the fundamentals of AI and its subdivisions.

## 3. Clinical Use Across the Transplant Pathway

### 3.1. Pre-Transplant Phase

#### 3.1.1. Organ Allocation and Donor–Recipient Matching

LT organ allocation depends on complex interactions among donor, recipient, perioperative, and postoperative factors. Applying ensemble methods to large, integrated datasets may improve organ allocation strategies and donor–recipient matching. For instance, a deep learning-based donor–recipient matching system has reported 95.8% overall accuracy in predicting post-transplant survival and 94.3% in forecasting in-hospital mortality [3]. Numerous studies show that artificial neural networks and random forests frequently outperform traditional statistical models in allocation tasks. Nevertheless, neural networks can be highly sensitive to data quality, and random forests may become computationally infeasible for very large datasets. A recent systematic review has identified numerous machine learning applications for donor–recipient matching, post-transplant survival prediction, and organ supply–demand modelling. Importantly, most studies focused on predicting post-transplant survival, whereas fewer robustly modelled transplant urgency, waitlist mortality, or net transplant benefit—the expected advantage of transplantation compared with remaining on the waiting list [4].

#### 3.1.2. Predicting Waiting-List Mortality and Disease Progression

Accurate waitlist mortality prediction is important because demand for donor livers exceeds supply. Although MELD-based scoring is central to liver allocation, it may not fully capture disease-specific, demographic, and longitudinal risk factors. AI and machine learning models can incorporate broader clinical histories and time-varying variables to estimate individual risk [5]. Neural network models trained on large transplant registries, such as United Network for Organ Sharing (UNOS)/Organ Procurement and Transplantation Network (OPTN), have outperformed MELD in selected cohorts, with improved sensitivity for identifying high-risk candidates [6]. More recently, the GEMA-AI (Gender-Equity Model for Liver Allocation using AI) was developed and validated in UK and Australian populations. It combines INR, bilirubin, sodium, and Royal Free GFR within an interpretable neural network and outperformed GEMA-Na and MELD-Na for waitlist mortality prediction, particularly in women and clinically complex cases [7]. Disease-specific ML models may add further value. A random survival forest for primary sclerosing cholangitis (PSC) outperformed MELD-Na and identified inflammatory markers, waiting time, platelet count, autoimmune hepatitis overlap, AST, sex, age, biliary interventions, and body weight as additional predictors [8]. In metabolic dysfunction-associated steatohepatitis cirrhosis, a DeepHit model analysed the competing risks of death and transplantation [9].

#### 3.1.3. Preoperative Functional Status Assessment

Candidate evaluation prior to LT is increasingly focusing on functional status, as cardiovascular complications and frailty significantly affect early and late post-transplant morbidity and mortality [10]. AI/ML offers opportunities to enhance personalised preoperative functional assessments and risk stratification. Key aspects include:

- **Conventional scoring systems (Table 1):** Traditional risk indices used for LT candidates, such as MELD, provide an objective framework for prioritising patients according to liver-disease severity [11]. However, MELD can underperform in certain conditions, including PSC, in which comorbid factors and disease-specific progression patterns are not fully captured. Other composite scores, such as the Survival Outcomes Following Liver Transplantation (SOFT) and Balance of Risk (BAR) scores, improve short-term outcome prediction—typically 90-day or 3-month patient and graft survival—but their calibration declines over longer follow-up. In an analysis of 112,357 transplants, these scores best predicted short-term patient and graft survival but performed poorly at 3- and 5-year follow-up [12].

- **Frailty and functional reserve assessment:** Frailty is a clinical condition reflecting reduced physiological reserve and is an established predictor of postoperative mortality and prolonged hospitalisation after LT [13]. The Liver Frailty Index, based on objective measures like grip strength, chair stands, and balance, can quantify pre-transplant frailty. A meta-analysis of 55 studies found that frailty prevalence in LT candidates ranged from 2.8% to 70.1% and that frail patients had significantly higher waiting-list and post-transplant mortality [14]. The Multidimensional Prognostic Index (MPI) is an alternative comprehensive geriatric assessment tool that may provide comparable prognostic value for older transplant candidates by incorporating nutrition, cognition, and activities of daily living (ADL) measures [15].
- **Sarcopenia assessment:** Sarcopenia is the generalised loss of skeletal muscle mass and strength and is commonly evaluated using imaging-derived measures such as the skeletal muscle index on abdominal CT or MRI. Preoperative sarcopenia is an important marker of frailty; in living donor LT, it independently predicts longer hospital and ICU stays and prolonged mechanical ventilation [16]. Recent deep learning techniques have enabled automated quantification of three-dimensional muscle volume from preoperative CT scans, producing results comparable to manual measurements with greater speed and consistency [17]. AI-driven sarcopenia assessment may improve risk stratification, identify candidates for prehabilitation or nutritional optimisation, and inform perioperative planning. Important barriers include standardising muscle-mass thresholds, accounting for scanner and acquisition variability, integrating tools with radiology image-viewing systems, and prospectively validating their clinical use.

### 3.2. AI in Donor Organ Assessment

#### 3.2.1. Current Challenges in Donor Liver Assessment

To expand the donor pool, many transplant programmes now consider expanded criteria donors, including older donors, steatotic (fatty) grafts, and organs donated after circulatory death (DCD). However, assessing marginal grafts is challenging: it is often subjective and relies on clinical judgement, a surgeon’s experience, and limited biomarker data [18]. The widely used Donor Risk Index (DRI) incorporates donor age, race, height, DCD vs. brain death donor (DBD), cause of death, split vs. whole graft, cold ischaemia time, and organ sharing, but it does not account for recipient factors or some extended donor variables [19]. Recipient variables that may refine AI/ML-based assessment include frailty, sarcopenia, cardiometabolic comorbidities, portal hypertension severity, renal dysfunction, inflammatory status, and previous abdominal surgery. Extended donor and graft variables may include macrovesicular steatosis, donor haemodynamic instability, vasopressor exposure, warm ischaemia time, preservation method, perfusate lactate clearance, bile production and pH, AST/ALT release, LDH, and other biochemical injury markers. AI/ML approaches may improve the objectivity and precision of donor liver evaluation by integrating complex data from donors, grafts, recipients, and dynamic perfusion platforms.

#### 3.2.2. AI/ML in Steatosis Assessment

Graft steatosis (fatty liver change) is a major reason for discarding donor livers and is associated with early allograft dysfunction, rejection, and worse long-term outcomes. Liver graft biopsy remains the gold standard for quantifying steatosis, but it is invasive and time-consuming; visual inspection of the liver is quicker but prone to variability and subjectivity. AI/ML techniques can support more standardised and reproducible assessment of steatosis:

- **Photographic and image-based analysis:** LiverColor is a recent platform combining colour and texture analysis of ex vivo graft photographs with supervised ML algorithms; it integrates donor characteristics, liver function tests, and graft images to estimate steatosis severity [20]. Additionally, near-infrared hyperspectral imaging coupled with one-dimensional convolutional neural networks (1D-CNNs) can classify the severity of steatosis and predict early allograft dysfunction. However, initial studies using this technology did not include paediatric donors, so further validation in broader cohorts is needed [21].
- **Histopathology via deep learning:** Foundation models such as Prov-GigaPath have been applied to quantify macrovesicular steatosis on frozen donor liver sections. In a recent study, this approach achieved 96.4% accuracy in categorising specimens above or below a 30% steatosis threshold [22]. While this method holds promise as a rapid, objective assessment of graft fat content, larger validation studies are required before routine clinical implementation.
- **MRI and advanced imaging modalities:** ML applied to magnetic resonance imaging (MRI)—specifically, measuring proton density fat fraction (PDFF)—allows non-invasive quantification of steatosis. This is particularly relevant in living donor LT, where pre-donation liver fat must be assessed without invasive biopsies. Convolutional neural networks (CNNs) and generative adversarial networks (GANs) have been investigated to improve PDFF accuracy, though current evidence is limited by small single-centre studies [23].

#### 3.2.3. Graft Viability and Biomarker Assessment

Machine perfusion is emerging as a promising enabling technology for graft assessment, converting preservation from a static process into a dynamic platform for repeated functional testing. Hypothermic oxygenated perfusion (HOPE), subnormothermic machine perfusion (SMP), and normothermic machine perfusion (NMP) differ in temperature, metabolic activity, and the data they generate, but all can provide richer information than static cold storage alone. NMP is particularly relevant to AI/ML because the liver is perfused under near-physiological conditions, enabling serial measurements of lactate clearance, bile production, bile pH and bicarbonate, glucose metabolism, perfusate AST/ALT, LDH, pH stability, vascular flows and resistance, and other markers like flavin mononucleotide, arginase-1, interleukin-6, and syndecan-1. These time-series variables can support models that forecast early allograft dysfunction, biliary complications, and transplantability more directly than pre-implantation clinical variables alone [24].

Clinical and translational evidence supports this broader role for NMP. The randomised COPE trial demonstrated that normothermic preservation reduced graft injury, lowered organ-discard rates, and extended preservation time compared with static cold storage [25]. The VITTAL trial further showed that NMP-based viability assessment enabled transplantation of 71% of livers previously declined by all UK centres, with viability judged primarily by lactate clearance to ≤2.5 mmol/L within 4 h and excellent early survival [26]. Longer-term NMP research has expanded the concept from viability testing toward an organ-support platform: Eshmuminov et al. showed that injured human livers could be preserved ex situ for up to 1 week with maintained function in selected grafts, and Clavien et al. reported successful transplantation after 3 days of ex situ normothermic perfusion [27,28]. Together, these studies suggest that NMP may extend the window for repeated diagnostics, therapeutic intervention, regeneration, and potential graft repair. Future AI/ML systems should therefore incorporate dynamic perfusion-derived biomarkers and their trends rather than treating machine perfusion as a single binary assessment.

### 3.3. Intraoperative Applications of AI/ML

#### 3.3.1. Intraoperative Complication Prediction

ML models have been used to forecast and manage surgical risks. For example, a CatBoost model has been trained to predict the need for massive transfusion during LT using preoperative and intraoperative variables, including patient age, haemoglobin, platelet count, coagulation parameters, and liver disease severity [29]. Similarly, the AI-OLTRBC-1 platform uses an ML-based approach that incorporates preoperative lab values (albumin, bilirubin, creatinine, haemoglobin, INR, and aPTT) to optimise blood product allocation and reduce transfusion delays [30]. Intraoperative ML models have also been developed to predict acute kidney injury (AKI) during LT; explainable ML approaches using Shapley Additive Explanations (SHAPs) have identified key risk factors (e.g., bilirubin, urine output, anaesthesia duration, platelet count, graft steatosis) to guide fluid management and improve perioperative decision-making [31].

#### 3.3.2. Surgical Guidance Applications

Beyond predictive analytics, AI and related technologies are being explored for surgical navigation. Mixed-reality systems (e.g., Magic Leap) can overlay 3D models of patient anatomy (such as liver vasculature) onto the surgical field to enhance intraoperative spatial awareness. In a study using a 3D-printed liver phantom, augmented reality (AR) guidance improved the precision of surgical resections, suggesting potential benefits for living donor or tumour resection procedures [32]. Another promising approach combines intraoperative ultrasound with laparoscopic or robotic visual cues. For example, Flag AR technology projects visual markers to highlight key anatomical structures (such as hepatic vessels) during minimally invasive donor hepatectomy, aiding surgeons in real time [33].

### 3.4. Postoperative Management

#### 3.4.1. Early Allograft Dysfunction (EAD) and Graft Failure Prediction

EAD is a serious complication linked to reduced graft survival and increased postoperative morbidity. The Liver Graft Assessment Following Transplantation (L-GrAFT) score is a validated model for predicting 90-day outcomes: it uses trends in AST, ALT, INR, bilirubin, platelet count, and creatinine over the first 7–10 post-transplant days to identify patients at risk of EAD and poor 3-month outcomes [34]. Additionally, novel biomarkers are under investigation: recent evidence suggests that neutrophil extracellular traps (NETs) contribute to early post-transplant complications through immune activation and thrombosis; these could be incorporated into future risk models [35].

#### 3.4.2. Detection of Acute Rejection

Acute rejection occurs in up to 30% of LT recipients within one year. AI-driven multimodal models are being developed to improve early diagnosis and characterisation of rejection. For example, by combining clinical variables with histopathology data, ML algorithms can classify T-cell-mediated rejections as steroid-responsive or steroid-resistant, which may guide therapy. Similarly, ML analysis of donor-derived cell-free DNA (dd-cfDNA) kinetics could improve rejection risk prediction, and CNN-based analysis of allograft biopsy slides can reduce inter-observer variability in applying the Banff classification for rejection severity [36].

#### 3.4.3. Immunosuppression Monitoring

AI/ML can support more objective, personalised immunosuppressive therapy after LT. Personalised dosing models integrate patient demographics, laboratory data, pharmacokinetic models, and time-series drug-level data to optimise tacrolimus dosing [37]. ML-based approaches have improved trough-level predictions and may reduce episodes of over- or under-immunosuppression. AI can also identify patterns of tacrolimus-associated nephrotoxicity, enabling earlier dose adjustments or conversion to mTOR inhibitor therapy [38]. Furthermore, ML has been used to stratify risk for opportunistic infections (e.g., cytomegalovirus disease or sepsis) using immune parameters and immunosuppressant exposure data. Outside the hospital, advanced monitoring tools such as wearable devices and “smart” medication dispensers can detect non-adherence and potentially predict increased rejection risk [39]. ML models may even anticipate long-term metabolic complications associated with chronic immunosuppressive therapy.

## 4. Implementation Challenges

Most AI research in transplantation has been retrospective and usually conducted at single centres. When models are applied to different populations or healthcare settings, their performance and calibration often decrease due to variations in donor demographics, patient groups, and clinical protocols. For AI to be effective in clinical practice, models need to report essential metrics like discrimination and calibration, and their real-world usefulness should be thoroughly tested—miscalibrated predictions, for instance, can negatively influence organ acceptance decisions or patient care. The main challenges include:

- **Data quality and heterogeneity:** High-quality, harmonised data remain a major barrier to robust AI. A review of transplant AI literature found that only approximately 6% of studies addressed algorithmic fairness and that 41% of ANN studies described no interpretability strategy [40]. Variation in data definitions, collection methods, and missingness across centres complicates model development and limits generalisability.
- **Lack of external validation:** Transplant populations, clinical practices, and allocation policies differ across regions and change over time. This “concept drift” can significantly decrease AI model performance on external datasets and emphasises the importance of ongoing retraining and validation [40]. Few AI models in LT have undergone prospective or multi-centre external validation, which is essential to prove real-world applicability.
- **Black-box models and explainability:** Many high-performing ML models (notably deep neural networks and ensemble methods) are complex and lack transparent decision pathways. This limited explainability can undermine clinician and patient trust in AI-generated risk estimates or treatment recommendations. The “black-box” problem of AI is driving efforts to incorporate explainable AI techniques (such as SHAP or LIME) to elucidate how models arrive at their predictions, thereby improving acceptance in clinical settings [41].
- **Organisational barriers:** Smaller transplant centres often lack the technical and financial resources needed to develop, implement, and sustain AI systems. Even highly accurate models must be integrated into existing workflows—such as electronic health records or organ-allocation systems—together with user training and robust data-governance infrastructure for model maintenance and updates. These organisational hurdles currently restrict broader adoption of AI in transplantation.

## 5. Ethical Considerations and Bias

AI in LT raises ethical concerns involving bias, transparency, accountability, patient privacy, and trust. Historical clinical data may encode disparities in listing practices, risk assessment, organ acceptance, and access to transplantation, which algorithms can reproduce or amplify. Underrepresentation of particular patient groups may also reduce model performance unless subgroup evaluation and fairness measures are incorporated into development and monitoring. Black-box models provide limited explanations for their predictions, making it difficult for clinicians and patients to understand or challenge AI-supported recommendations. Responsibility for decisions must therefore remain clearly assigned, with defined escalation and override processes. Safe use requires comprehensive bias monitoring, transparent reporting of negative results and errors, strict privacy protections, meaningful patient involvement, and continuing human oversight.

## 6. Conceptual Framework for Responsible AI Deployment

Safe and effective AI deployment in liver transplantation requires a structured, responsible AI framework. Current decision-making often relies on a sequential review of clinical judgement, donor risk scores, recipient urgency, visual graft assessment, static laboratory values, and centre-specific experience. Future AI-enabled decision support should instead integrate these inputs with dynamic imaging, pathology, perfusion-derived biomarkers, longitudinal electronic health records, and explainable risk estimates that are continuously recalibrated after deployment. The goal is not automated organ acceptance or rejection, but an auditable comparison of current practice with model-assisted forecasting of graft viability, waitlist urgency, perioperative risk, and post-transplant complications. Key principles include data quality, completeness, validation, interpretability, calibration, clinician oversight, equity monitoring, and patient safety. A multidisciplinary governance approach is essential and should involve transplant clinicians, data scientists, ethicists, regulators, and patient representatives. This ensures that AI supports—rather than supplants—clinical judgement. Figure 2 illustrates a stepwise approach to implementing AI in LT while adhering to these principles. Table 2 summarises major AI/ML models used in liver transplantation, their reported predictive performance, and clinical utility.

## 7. Future Directions and Enabling Technologies

### 7.1. Multimodal Data Integration

Future predictive models for LT should integrate multiple data types, including genomic, immunologic, proteomic, imaging, clinical, and intraoperative parameters. Combining these data into single models may improve the accuracy and personalisation of risk predictions for transplant outcomes. However, this approach depends on well-curated, standardised datasets and thorough validation across large, diverse patient cohorts.

### 7.2. Generative AI and Large Language Models

Generative AI tools, such as large language models (LLMs), can help clinicians by summarising unstructured clinical data (such as narrative notes) and supporting complex reasoning or decision-making. However, their use in transplantation remains largely theoretical. Careful assessment is essential to confirm that these models provide accurate, unbiased information and integrate smoothly into clinical workflows without compromising patient safety or care quality.

### 7.3. Federated Learning and Explainable AI Models

Federated learning is a growing approach that enables multiple institutions to collaboratively develop AI models using local data without sharing sensitive patient information across borders. This approach can support robust multi-centre AI systems while maintaining data privacy. Combining federated learning with explainability features or model-agnostic explanation techniques may also increase clinicians’ confidence and improve the interpretability of predictions, which is essential for clinical use.

### 7.4. Machine Perfusion and AI/ML-Enabled Assessment

Machine perfusion is now playing an important role in organ preservation. Both normothermic and hypothermic perfusion produce a continuous stream of biomarker data, and AI/ML models can combine this data with information on the donor, the graft, the recipient, imaging results, and histopathology in order to improve the evaluation of graft viability, the risk of early dysfunction, the risk of cholangiopathy, and the suitability of the transplant [24]. In the future, explainable ML tools incorporated into the perfusion processes could help with real-time accept-or-decline decision-making and lead to a standardisation of viability assessments in different transplant centres. However, such systems will need prospective validation, data standardisation, and clinical supervision before they can be safely introduced into routine practice.

## 8. Recommendations for Implementation

### 8.1. Rigorous Validation

AI models must undergo prospective and external validation across multiple transplant centres and patient groups. When presenting new models, report their performance metrics for relevant subgroups, including donor type, recipient demographics, and disease aetiology. Additionally, assess their calibration over time to verify consistent reliability.

### 8.2. Governance and Ethics

Implementing AI in transplantation requires a robust governance framework that includes stakeholders from clinical, data science, regulatory, and patient communities. This promotes ethical practices and transparency throughout development and deployment. It is also crucial to establish accountability and clear guidelines for the use of AI outputs in clinical decision-making.

### 8.3. Clinician and Patient Engagement

To facilitate adoption, AI systems should be developed with direct input from end-users. User-friendly interfaces and integration with existing clinical decision support tools are important. Clinicians and patients must be engaged in understanding how the AI’s recommendations are generated and their limitations, especially for sensitive decisions such as organ allocation or changes in post-transplant management.

## 9. Conclusions

AI and ML hold great promise to improve donor–recipient matching, immunosuppression control, and outcome forecasting in liver transplantation. To turn this potential into practice, careful implementation is needed: validation across different settings, ongoing calibration and updates, and strong clinician oversight to ensure patient safety and preserve trust. Progress in explainable AI, federated learning, and multimodal data integration supports responsible AI use in this field. Ultimately, using these technologies to support, not replace, clinician judgement can enhance personalised care and outcomes, as long as patient-centeredness, equity, and ethics continue to be prioritised.

## Figures and Tables

**Figure 1:**
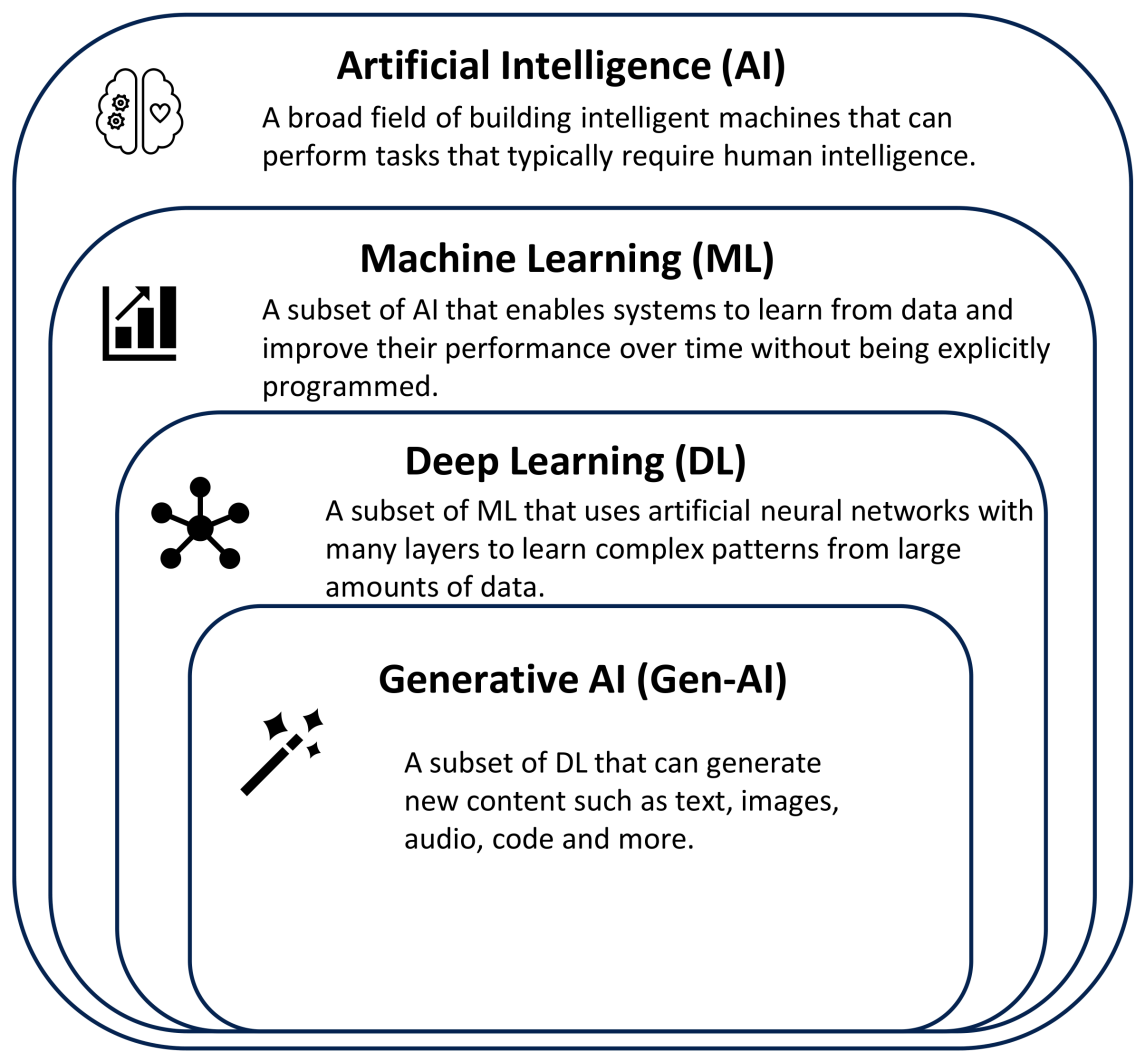
Fundamental concepts of artificial intelligence.

**Figure 2:**
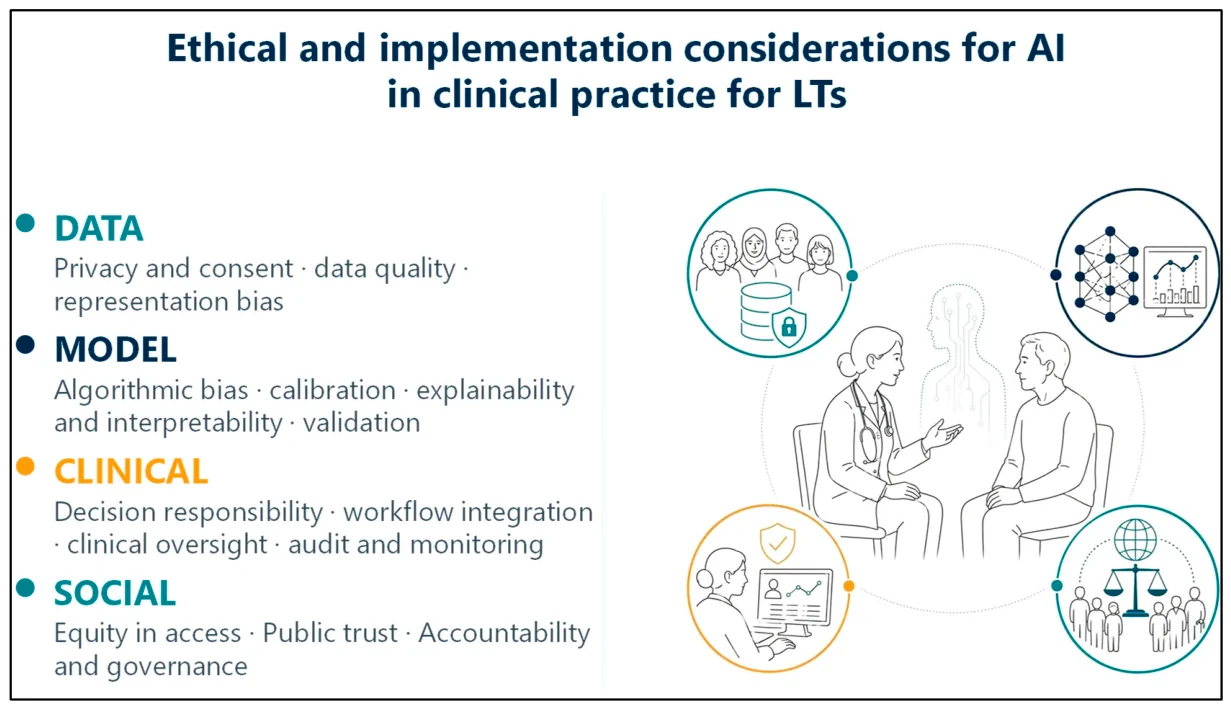
A stepwise approach to implementing AI in LT while adhering to these principles.

**Table 1:** Conventional scoring systems in liver transplantation.

Score	Variables Included	Reference/Normal Ranges	Clinical Use
MELD [11]	Serum bilirubin, INR, serum creatinine (dialysis adjustment)	Bilirubin 0.3–1.2 mg/dL; INR 0.8–1.2; Creatinine 0.6–1.2 mg/dL; MELD score range: 6–40	Predicts 90-day mortality; primary tool for liver transplant allocation
MELD-Na [11]	MELD + serum sodium	Sodium 135–145 mmol/L (score caps at 125–137 mmol/L)	Improves mortality prediction in hyponatraemic patients; standard for transplant prioritisation
MELD 3.0 [11]	MELD + albumin + sex coefficient	Albumin 3.5–5.0 g/dL; includes female sex coefficient	Reduces sex-based disparities; improves prediction, especially in women and patients with preserved albumin levels
SOFT [12]	Recipient age, MELD, life support requirement, previous transplant, portal vein thrombosis, donor age, cause of death, cold ischaemia time	Score ≥ 12 is associated with increased 3-month mortality	Predicts early post-transplant survival; supports donor–recipient matching decisions
BAR [12]	Recipient age, MELD, retransplantation, life support, donor age, cold ischaemia time	Score range 0–27; BAR < 18 indicates low risk	Predicts 90-day post-transplant mortality; used for risk stratification and to assess transplant suitability

Note: Laboratory reference ranges vary by institution and assay; the values shown are representative adult clinical ranges. Score cut-offs and clinical uses are summarised from validation and comparative studies of MELD, MELD-Na, MELD 3.0, SOFT, and BAR rather than from a single source [11,12]. These ranges should not be interpreted as universal decision thresholds.

**Table 2:** Selected AI/ML models studied in liver transplantation and related clinical utility.

Clinical Area	Model/Approach	Reported Performance	Clinical Utility and Limitations
Donor–recipient matching	Deep learning donor–recipient matching system.	Overall accuracy 95.8% for post-transplant survival prediction; 94.3% for in-hospital mortality prediction [3].	May support matching and allocation decisions, but requires external validation, calibration, and transplant-benefit modelling.
Waitlist mortality	Neural networks; GEMA-AI; disease-specific random survival forests and DeepHit models.	Several models outperform MELD or MELD-Na in selected cohorts [6,7,8,9].	Potential to improve prioritisation and identify high-risk candidates; prospective validation remains essential.
Steatosis assessment	Photographic ML, hyperspectral imaging with CNNs, and foundation pathology models.	A foundation pathology model reported 96.4% accuracy for classifying above or below a 30% macrovesicular steatosis threshold [22].	Could standardise graft fat assessment and reduce subjectivity; paediatric and multicentre validation are limited.
Machine perfusion viability	ML integration of perfusate lactate, bile metrics, transaminases, LDH, flow and resistance trends.	Early reports suggest potential for EAD prediction, but robust sensitivity, specificity, PPV and NPV remain inconsistently reported [24].	Promising for transplant-versus-discard decisions during NMP; substantial prospective validation is needed.
Intraoperative risk	CatBoost and explainable supervised ML models.	Models have predicted massive transfusion and AKI using perioperative variables [29,30,31].	May guide blood product planning, renal-protective strategies, and intraoperative monitoring; integration into workflow is needed.
Immunosuppression	Pharmacokinetic ML models for tacrolimus concentration and nephrotoxicity prediction.	Retrospective cohorts have reported improved trough-level prediction and nephrotoxicity risk stratification [38].	This could individualise dosing and monitoring, but it should be paired with clinician review and therapeutic drug monitoring.

## Data Availability

No new data were created or analysed in this study. Data sharing is not applicable to this article.

## Abbreviations

The following abbreviations are used in this manuscript:

ADL	Activities of daily living
AI	Artificial intelligence
AI/ML	Artificial intelligence/machine learning
AKI	Acute kidney injury
ALT	Alanine aminotransferase
ANN	Artificial neural network
aPTT	Activated partial thromboplastin time
AR	Augmented reality
AST	Aspartate aminotransferase
BAR	Balance of Risk
CNN	Convolutional neural network
CT	Computed tomography
DBD	Donation after Brain Death
DCD	Donation after circulatory death
dd-cfDNA	Donor-derived cell-free DNA
DRI	Donor Risk Index
EAD	Early allograft dysfunction
EHR	Electronic health record
GEMA-AI	Gender-Equity Model for Liver Allocation using Artificial Intelligence
GFR	Glomerular filtration rate
HPB	Hepatopancreatobiliary
HOPE	Hypothermic oxygenated perfusion
ICU	Intensive care unit
INR	International normalised ratio
L-GrAFT	Liver Graft Assessment Following Transplantation
LDH	Lactate dehydrogenase
LIME	Local Interpretable Model-Agnostic Explanations
LLM	Large language model
LT	Liver transplantation
MASH	Metabolic dysfunction-associated steatohepatitis
MELD	Model for End-Stage Liver Disease
ML	Machine learning
MPI	Multidimensional Prognostic Index
MRI	Magnetic resonance imaging
mTOR	Mechanistic target of rapamycin
NASH	Non-alcoholic steatohepatitis
NET	Neutrophil extracellular trap
NMP	Normothermic machine perfusion
OPTN	Organ Procurement and Transplantation Network
PACS	Picture archiving and communication system
PDFF	Proton density fat fraction
PSC	Primary sclerosing cholangitis
SHAP	Shapley Additive Explanations
SMP	Subnormothermic machine perfusion
SOFT	Survival Outcomes Following Liver Transplantation
UNOS	United Network for Organ Sharing
